# Understanding implementation research

**DOI:** 10.4102/phcfm.v17i2.4934

**Published:** 2025-06-03

**Authors:** Robert Mash, Juliet Nyasulu, Zelra Malan, Lisa Hirschhorn

**Affiliations:** 1 Division of Family Medicine and Primary Care, Faculty of Medicine and Health Sciences, Stellenbosch University, Cape Town, South Africa; 2 Division of Health Systems and Public Health, Faculty of Medicine and Health Sciences, Stellenbosch University, Cape Town, South Africa; 3 Division of Family Medicine, University of Namibia, Windhoek, Namibia; 4 Department of Medical Social Sciences, Feinberg School of Medicine, Northwestern University, Chicago, United States

**Keywords:** implementation, implementation research, methodology, methods, implementation outcomes, implementation strategies, primary care

## Abstract

Implementation research (IR) focuses on understanding and closing the gap between evidence-based interventions and practice. Key elements to evaluate include the design of the intervention itself, contextual barriers and enablers to implementation, the use of implementation strategies as well as the achievement of implementation outcomes. This article gives an overview of IR for doctoral-level researchers in the fields of family medicine and primary care. The consolidated framework for IR and socioecological model are considered for making sense of the contextual factors. A typology of implementation strategies is also described to make conceptualisation, reporting and sharing of findings easier. Standard implementation outcomes are described, such as coverage or reach, acceptability, adoption, appropriateness, feasibility, fidelity, costs and sustainability. The RE-AIM framework for implementation outcomes is described. Finally, different study designs are discussed, including hybrid effectiveness-implementation designs and approaches to reporting using the IR logic model.

## Introduction

Basic research develops new products, technologies or processes and has given health care a plethora of interventions known to reduce suffering and save lives.^1^ Evidence-based medicine has focused on whether these interventions could work under ideal conditions in efficacy studies or in the real world in effectiveness studies.^2^ However, over decades, there has been a substantial gap between having an evidence-based intervention and implementing it with quality, equity and at scale in the health system.^3^

Implementation research (IR) studies look at understanding and addressing challenges of translating evidence-based interventions and policies into real-world practice within health systems.^4^ This research often involves multidisciplinary approaches and participatory methods to ensure relevance and applicability in diverse settings and examines the processes, contextual factors and outcomes associated with the adoption, implementation and sustainability of health interventions, policies or programmes within specific health systems or communities.^5^ A lack of implementation of generated evidence reduces the potential quality of care and utilisation of evidence-based interventions and increases the number of avoidable deaths.^6^ This is true across the burden of disease from noncommunicable diseases to HIV and tuberculosis, maternal and neonatal health, mental health and physical trauma.

This focus on improving the quality of care highlights an overlap of IR with quality improvement.^4^ Quality improvement processes are usually more focused on the local or facility level, while IR is concerned with generalisable or transferable knowledge. Knowledge translation also can overlap with components of IR but is usually more focused on how to communicate evidence to policymakers and influence policy. Similarly, science communication focuses more on informing and engaging the general public or nonexpert audiences. Knowledge translation and IR can be brought together by involving or embedding policy- and decision-makers in the research team.^7^ Translational research is another related concept that primarily focuses on the journey from ‘bench to bedside’ and how basic science derived in the laboratory can be converted into useful tools and applications for humans.

Most health problems are managed in primary care, and African family medicine also includes care at the primary hospital.^8^ In this context, the focus is often on improving the quality of service delivery and patient safety by successfully implementing what we already know is effective. Closing the knowledge–practice gap is a particular research interest for family medicine and primary care researchers.^9^ Therefore, this article aims to highlight the role of IR in improving primary health care and family medicine.

## The need for implementation research

The toolkit of evidence-based interventions continues to grow globally, and with it, the knowledge–practice gap.^10^ Some interventions are not implemented at all or are implemented with poor fidelity to the design that was shown to be effective. Some interventions only benefit high-income countries or the more affluent and increase inequity, while others may be implemented in a limited way that does not go to scale across the whole population at risk. On average, it can take up to 17 years to successfully implement an evidence-based intervention, and only 14% of interventions reach their target group.^11,12^ As a result, primary care providers and family medicine practitioners do not have the tools needed to improve outcomes, and implementation needs to be a focus for researchers in these fields.

Some of the plausible reasons for evidence-based interventions not working in the real world could be:^13,14,15^

- the intervention was the wrong one for the population (e.g. implementing individual brief behaviour change counselling instead of group empowerment for people with type 2 diabetes),
- the intervention was not delivered as planned (e.g. key components of the intervention were not implemented, such as the wrong hypertension medications),
- the wrong strategies were used to implement the intervention (e.g. over-reliance on training healthcare workers and not engaging decision makers),
- the correct strategies were poorly implemented (e.g. the wrong people sent to the training course, supportive supervision was done solely as audit), and
- previously existing barriers (e.g. cultural distrust, weak supporting systems) or new barriers (e.g. coronavirus disease 2019 [COVID-19] pandemic) could not be overcome by the chosen strategies.

Finding answers on how to prevent or address all these gaps and getting the right care to the right people can be addressed by IR.

## The key elements of implementation research

Implementation research deconstructs the process of implementation and enables the different elements to be studied. Four different elements can be considered:^16^

1. The intervention: the design of the intervention and the strength of the evidence that it is effective.
2. Contextual factors: factors in the context that may act as barriers or enablers to implementation.
3. Implementation strategies: the actions that we take to help people or systems use the intervention.
4. Implementation outcomes: outcomes that can be evaluated to determine whether implementation is effective.

Each of these elements has its own concepts and frameworks that will be considered below. Frameworks can help provide insights into contextual factors, help with planning and guide evaluation (prospective or retrospective).^16^

### The intervention

Typically, IR begins once an evidence-based intervention has been established as effective and needs to be implemented. However, interventions are often being implemented with an evidence base that is still incomplete. There may be a need to design studies that simultaneously evaluate the effectiveness of the intervention and its implementation. This could mean evaluating intervention effectiveness in terms of health outcomes and implementation effectiveness in terms of implementation outcomes at the same time. These may be referred to as hybrid effectiveness-implementation trials, as shown in Table 1.^17,18,19^

**TABLE 1:** Hybrid effectiveness-implementation trial designs.^17,18,19^

Hybrid design type	Description
Type 1: Clinical intervention with implementation observation	Testing the effects of a clinical intervention on relevant outcomes while observing and gathering information on implementation.
Type 2: Dual testing of clinical and implementation interventions	Simultaneously testing both clinical and implementation interventions or strategies.
Type 3: Implementation strategy with clinical observation	Testing an implementation strategy while observing and gathering information on the clinical intervention’s impact on relevant outcomes.

Note: Table 1 was adapted from references^17,18,19^ found in the reference list of this article, Mash R, Nyasulu J, Malan Z, Hirschhorn L. Understanding implementation research. Afr J Prm Health Care Fam Med. 2025;17(2), a4934. https://doi.org/10.4102/phcfm.v17i2.4934, for more information.

When reporting on IR, it is important to fully describe the design of the intervention. Key issues to consider include the development of the intervention and the strength of the evidence for its effectiveness. The complexity of the intervention may also be an issue as well as the design quality and packaging.

### Contextual factors

**FIGURE 1:**
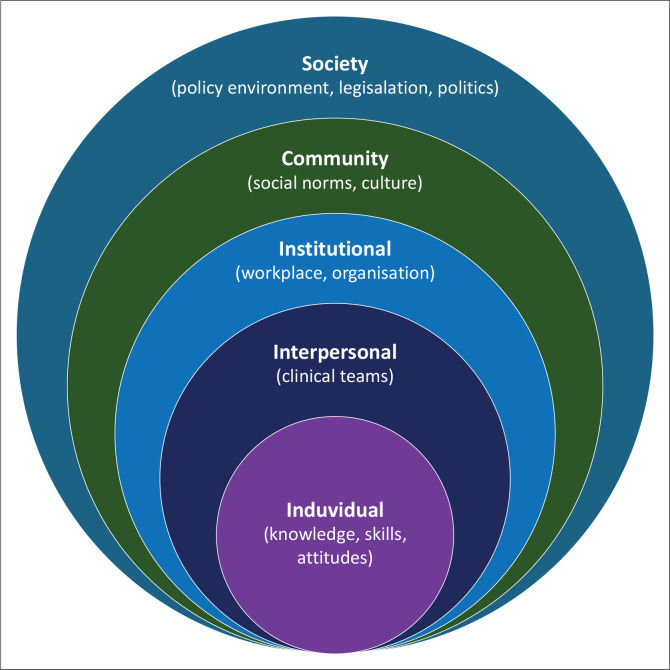
The socioecological model.

Before designing and testing implementation, it may be necessary to understand the context in more detail. These contextual barriers and enablers can be conceptualised by determinant frameworks. There are many frameworks such as the Consolidated Framework for Implementation Research (CFIR) 1.0, the socioecological model and theoretical domains.^16^ The CFIR has five domains to consider, as shown in Table 2, and three of them refer directly to the contextual factors: outer setting, inner setting and individual characteristics. The socioecological model similarly considers the context of implementation as a series of nested systems putting the individual in the centre. Moving from the immediate individual to the broader society as shown in Figure 1.^20^

**TABLE 2:** Domains of the consolidated framework for implementation research.^16^

Domain	Description
Innovation characteristics	The characteristics of the intervention itself. Its design, source, evidence base, complexity and packaging.
Outer setting	The economic, political, policy and social context within which the implementing organisation resides.
Inner setting	The structure, culture, goals, resources, relationships and leadership of the implementing organisation.
Individual characteristics	Knowledge, skills, motivation, attributes and beliefs of the people that are expected to implement the intervention.
Process	Planning implementation and adapting intervention, engaging decision-makers and individuals in processes, executing the plan, evaluating progress.

*Source*: Adapted from Rapport F, Clay-Williams R, Braithwaite J. Implementation science: The key concepts. Abingdon: Routledge, 2022; 236 p.

Contextual factors identified in mixed methods research can be categorised and better understood by using these frameworks. Often the same factor could be both a barrier and an enabler depending on how strongly it is present.

### Implementation strategies

**FIGURE 2:**
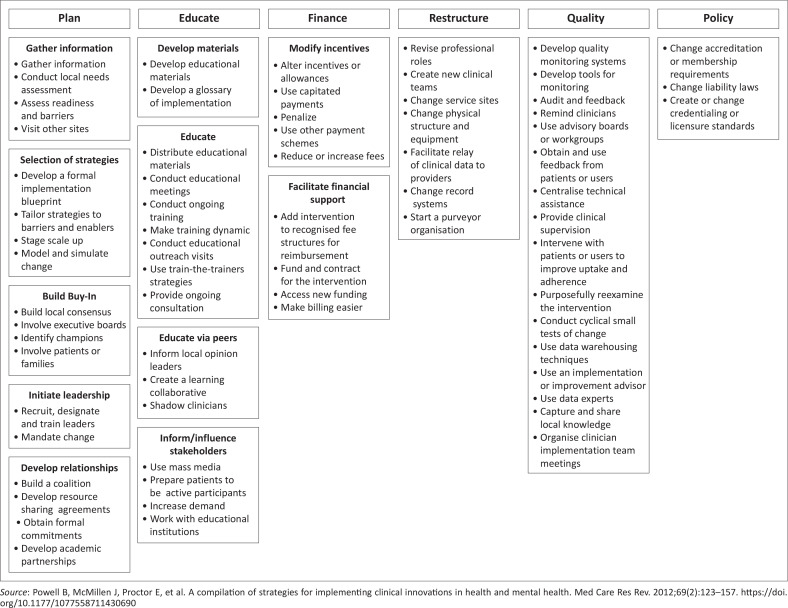
Typology of implementation strategies.^22^

Interventions are often implemented with little thought given to the range of strategies and how they can be conceptualised. Strategies are often implicit and poorly defined. It is helpful to have a standardised way of labelling and categorising strategies.^21^ This not only improves understanding of how an intervention is being implemented but also enables researchers to share a common language and compare findings. Figure 2 presents one of the commonly used typologies of implementation strategies.^22^ Strategies are organised into six domains related to planning, education, finances, restructuring, quality improvement and the policy context.

Strategies have also been organised into nine categories as expert recommendations for implementing change (ERIC):^23^

1. Engage consumers or users
2. Use evaluative and iterative strategies
3. Change infrastructure
4. Adapt and tailor to the context
5. Develop stakeholder interrelationships
6. Utilise financial strategies
7. Support clinicians
8. Provide interactive assistance
9. Train and educate stakeholders

Implementation strategies can be designed to consciously address the barriers and leverage the enablers as illustrated in Table 3. In addition, publications on documenting strategies in low- and middle-income countries have identified strategies not captured in ERIC. These were predominantly strategies targeting systems- or policy-level barriers and highlight the need for additional research and conceptualisation of strategies in these settings.^24^

**TABLE 3:** Examples of strategies designed to address barriers to implementation.

Identified barriers	Relevant implementation strategies
Lack of knowledge	Conduct educational meetings or ongoing training
Slow implementation	Audit and feedback
Lack of motivation	Provide incentives or penalise nonadherence Provide clinical supervision Audit and feedback
Community beliefs or attitudes to intervention	Inform and engage local opinion leaders Use mass media

### Implementation outcomes

We are used to thinking about clinical outcomes in terms of improved health status or symptoms for a particular disease, functioning or quality of life. Cross-cutting health service outcomes look at issues such as safety, efficiency, equity, timeliness and the core functions of primary care: first contact access, coordination, continuity, comprehensiveness and person centredness. Implementation outcomes are a different set of outcomes from what we are used to. Eight commonly used implementation outcomes are listed in Table 4.^25^

**TABLE 4:** Examples of implementation outcomes.^25^

Implementation outcomes	Working definition
Coverage or reach	Degree to which an eligible or targeted population receives the intervention
Acceptability	Perception among stakeholders that an intervention is acceptable, and they are willing to participate or access it
Adoption	Factors influencing the institutional decision to adopt or support given by institution to implement
Appropriateness	Perceived fit of the intervention in a particular setting for a particular population
Feasibility	Extent to which an intervention can be carried out in a particular setting or a person can deliver or use it
Fidelity	Degree to which an intervention was implemented as it was designed in an original protocol, plan or policy or after adaptation
Costs	Incremental or opportunity costs of strategies to deliver the intervention
Sustainability	Extent to which an intervention is institutionalised or the impact is sustained in individuals

*Source*: Adapted from Proctor E, Silmere H, Raghavan R, et al. Outcomes for implementation research: Conceptual distinctions, measurement challenges, and research agenda. Adm Policy Ment Health. 2011;38(2):65–76.

**FIGURE 3:**
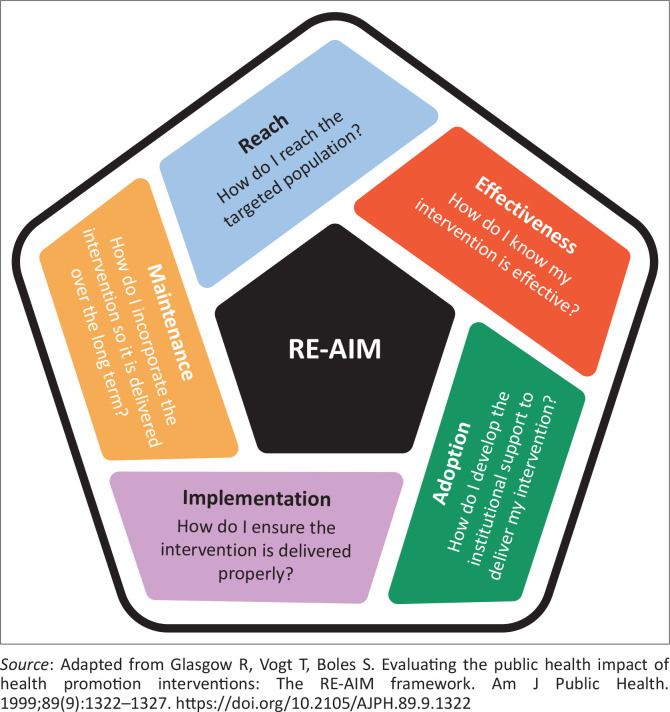
RE-AIM framework for evaluating implementation outcomes.^26^

Frameworks have also been developed to help measure implementation outcomes. The most common is the RE-AIM framework as shown in Figure 3.^26^ The one element that requires explanation is that of effectiveness. In this context, the intention is not usually to prove the effectiveness of the intervention as one might do in a clinical trial but to ensure that the expected effects of the intervention are happening. Of course, hybrid effectiveness-implementation trials might combine the two objectives, but often the focus is more on the effects than on effectiveness per se.

To evaluate sustainability, there would need to be a reasonable period between implementation and evaluation. This allows the researcher to evaluate whether the intervention continues and individual behaviour is maintained, whether the intervention has evolved and adapted and whether it continues to have beneficial effects. Sustainability is often a problem in low-resourced settings. Too often, researchers introduce and evaluate interventions over a short period that is aligned with funding cycles and the need for research outputs. Once the funding and researcher disappear, so does the intervention. Health systems and beneficiaries are more interested in longer-term benefits and returns on investment.

## Implementation research questions

Research questions would focus on one or more of the key concepts described above. For example, exploring the contextual factors prior to implementation to plan a better intervention and implementation strategies. Often studies focus on evaluating the implementation outcomes using a framework such as RE-AIM and might also identify the key contextual factors influencing these outcomes. Studies could also evaluate different implementation strategies and the mechanisms by which they lead to outcomes. For example: Does training nurses at lower level facilities to identify patients and prescribe hypertensive treatment as part of routine care lead to provider adoption and to reaching more eligible patients compared to referring patients to higher level facilities?

This research question includes both implementation strategies (training and restructuring care), an intervention (hypertensive treatment) and implementation outcomes (adoption and reach).

## Study designs for implementation research

Implementation science is the study of methods to promote the integration of research findings into healthcare practice. There are no unique methods to IR, but often a mixed-methods approach is needed. For example, in evaluating implementation outcomes, there may be quantitative data to measure reach, costs and effects, but qualitative data to explore acceptability, appropriateness, adoption, feasibility and fidelity. The usual methodological considerations for collecting and analysing such data would apply. Descriptive exploratory qualitative studies, observational and experimental studies (e.g. before-and-after, clinical trials) and mixed methods are all possibilities.^27^

Embedded approaches to research are common.^7^ For example, the researchers may work within the healthcare system and be part of implementation or may actively engage stakeholders such as policymakers, practitioners or community members in the research process. Participatory action research may be an appropriate methodology as participants seek to change their reality while also researching and learning from their experience. All these approaches can collaboratively enhance our understanding of implementation.

Hybrid implementation-effectiveness studies were described earlier. Newer and more complex designs may also be valuable, such as stepped wedge or adaptive platform clinical trials. These could potentially allow multiple strategies to be tested consecutively with sample sizes determined by achieving probability thresholds.

## Reporting and disseminating implementation research results

The four key elements discussed in this article should be included in reporting on IR. For example, if the focus of the study is on evaluating implementation outcomes and identifying the key determinants that influenced these outcomes, then the methods section should contain a detailed description of the intervention and in a separate section the implementation strategies. The findings will report on the contextual factors and the outcomes.^28^ The StaRI (Standards for Reporting Implementation Studies) statement provides a checklist that focuses on reporting the evaluation of implementation strategies.^28^ This statement may not be applicable to all IR studies.

**FIGURE 4:**
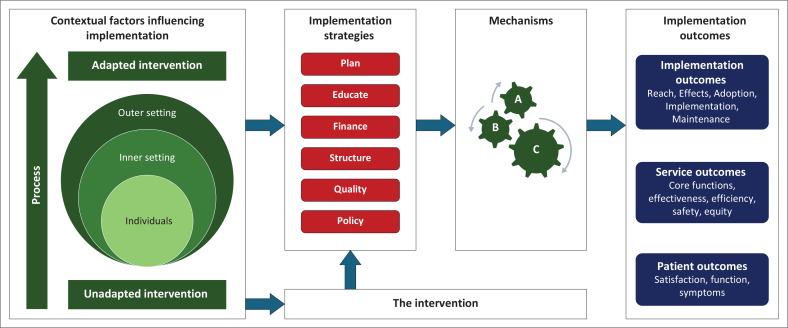
Template for the implementation research logic model.

**FIGURE 5:**
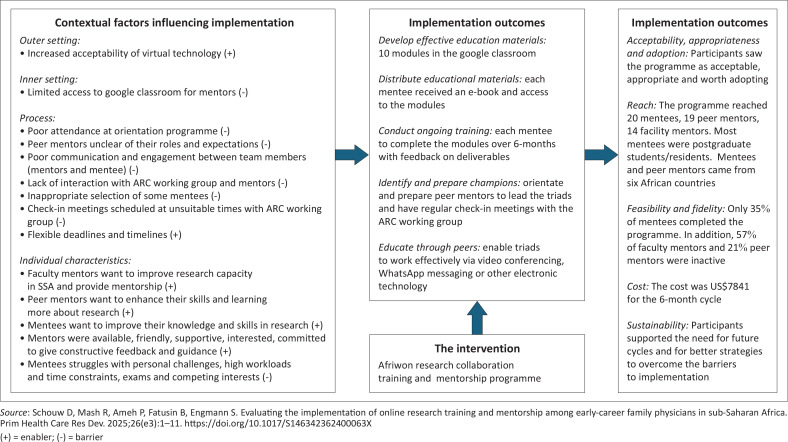
Example of an implementation research logic model.^30^

The implementation research logic model (IRLM) (Figure 4) can be a useful framework for summarising all the findings and key elements and showing their relationships.^29^ The contextual factors or determinants can be listed using the CFIR, the strategies according to their typology and the outcomes that are relevant to the study. The mechanisms by which the strategies achieved the outcomes can also be considered. An example of an IRLM that was used to summarise the implementation of an e-mentorship programme for novice researchers in sub-Saharan Africa is shown in Figure 5.^30^

## Conclusion

This series looks at methods for doctoral-level and early career researchers in the fields of family medicine and primary care. Implementation research could be the focus of your doctoral degree or one of the studies within it. Implementation research is a growing field, and there are a plethora of frameworks and concepts that are too many to present here. Being familiar with the key IR elements and a few of the frameworks can assist you to design and to report on your study. This should help you to position yourself in the field and communicate what you have done and found in a way that other implementation researchers can understand, review and relate to.

## References

[1] National Science Board. Science and Engineering Indicators [homepage on the Internet]. Alexandria, VA; 2018. [Cited 2025 February 17]. Available from: https://www.nsf.gov/statistics/indicators/

[2] Singal A, Higgins P, Waljee A. A primer on effectiveness and efficacy trials. Clin Transl Gastroenterol. 2014;5(1):e45. 10.1038/ctg.2013.13 24384867 PMC3912314

[3] Kruk M, Pate M. The Lancet Global Health Commission on high quality health systems 1 year on: Progress on a global imperative. Lancet Glob Health. 2020;8(1):e30–e32. 10.1016/S2214-109X(19)30485-1 31839136

[4] Peters DH, Adam T, Alonge O, Agyepong IA, Tran N. Implementation research: What it is and how to do it. BMJ. 2013;347:f6753. 10.1136/bmj.f6753 24259324

[5] Peters D, Tran N, Adam T. Implementation research in health: A practical guide. Geneva: World Health Organization; 2013.

[6] Amberbir A, Sayinzoga F, Mathewos K, et al. Maintaining delivery of evidence-based interventions to reduce under-5 mortality during COVID-19 in Rwanda: Lessons learned through implementation research. Ann Glob Health. 2024;90(1):47. 10.5334/aogh.4348 39070076 PMC11276474

[7] Biermann O, Schleiff M, Romao D, Mikaelsdotter C, Alfven T, Wanyenze R. Action towards connecting knowledge translation and implementation research. Lancet Glob Health. 2025;13(3):e404. 10.1016/S2214-109X(24)00522-9 39929226

[8] Mash R. The contribution of family physicians to African health systems. Afr J Prim Health Care Fam Med. 2022;14(1):a3651. 10.4102/phcfm.v14i1.3651 PMC9350474 35924626

[9] Nazha B, Yang J, Owonikoko T. Benefits and limitations of real-world evidence: Lessons from EGFR mutation-positive non-small-cell lung cancer. Future Oncol. 2021;17(8):965–977. 10.2217/fon-2020-0951 33242257

[10] Boaz A, Baeza J, Fraser A, Persson E. ‘It depends’: What 86 systematic reviews tell us about what strategies to use to support the use of research in clinical practice. Implement Sci. 2024;19(1):15. 10.1186/s13012-024-01337-z 38374051 PMC10875780

[11] Balas E, Boren S. Managing clinical knowledge for health care improvement. Yearb Med Inform. 2000;9(1):65–70. 10.1055/s-0038-1637943 27699347

[12] Green L, Glasgow R, Atkins D, Stange K. Making evidence from research more relevant, useful, and actionable in policy, program planning, and practice: Slips ‘twixt cup and lip’. Am J Prev Med. 2009;37(6Suppl1):S187–S191. 10.1016/j.amepre.2009.08.017 19896017

[13] Klaic M, Kapp S, Hudson P, et al. Implementability of healthcare interventions: An overview of reviews and development of a conceptual framework. Implement Sci. 2022;17(1):10. 10.1186/s13012-021-01171-7 35086538 PMC8793098

[14] Thomas A, Ellaway R. Rethinking implementation science for health professions education: A manifesto for change. Perspect Med Educ. 2021;10(6):362–368. 10.1007/S40037-021-00688-3 34757538 PMC8633355

[15] Peters-Corbett A, Parke S, Bear H, Clarke T. Barriers and facilitators of implementation of evidence-based interventions in children and young people’s mental health care – A systematic review. Child Adolesc Ment Health. 2023;29(3):242–265. 10.1111/camh.12672 37608642

[16] Rapport F, Clay-Williams R, Braithwaite J. Implementation science: The key concepts. Abingdon: Routledge, 2022; 236 p.

[17] Curran G, Bauer M, Mittman B, Pyne J, Stetler C. Effectiveness-implementation hybrid designs: Combining elements of clinical effectiveness and implementation research to enhance public health impact. Med Care. 2012;50(3):217–226. 10.1097/MLR.0b013e3182408812 22310560 PMC3731143

[18] Landes S, McBain S, Curran G. An introduction to effectiveness-implementation hybrid designs. Psychiatry Res. 2019;280. 10.1016/j.psychres.2019.112513 31434011 PMC6779135

[19] Ullman A, Beidas R, Bonafide C. Methodological progress note: Hybrid effectiveness-implementation clinical trials. J Hosp Med. 2022;17(11):912–916. 10.1002/jhm.12936 35934981 PMC9804495

[20] Bronfenbrenner U. Toward an experimental ecology of human development. Am Psychol. 1977;32(7):513–531. 10.1037/0003-066X.32.7.513

[21] Proctor E, Powell B, McMIllen J. Implementation strategies: Recommendations for specifying and reporting. Implement Sci. 2013;8:139. 10.1186/1748-5908-8-139 24289295 PMC3882890

[22] Powell B, McMillen J, Proctor E, et al. A compilation of strategies for implementing clinical innovations in health and mental health. Med Care Res Rev. 2012;69(2):123–157. 10.1177/1077558711430690 22203646 PMC3524416

[23] Powell B, Waltz T, Chinman M, et al. A refined compilation of implementation strategies: Results from the Expert Recommendations for Implementing Change (ERIC) project. Implement Sci. 2015;10:21. 10.1186/s13012-015-0209-1 25889199 PMC4328074

[24] Lovero K, Kemp C, Wagenaar B, et al. Application of the Expert Recommendations for Implementing Change (ERIC) compilation of strategies to health intervention implementation in low- and middle-income countries: A systematic review. Implement Sci. 2023;18(1):56. 10.1186/s13012-023-01310-2 37904218 PMC10617067

[25] Proctor E, Silmere H, Raghavan R, et al. Outcomes for implementation research: Conceptual distinctions, measurement challenges, and research agenda. Adm Policy Ment Health. 2011;38(2):65–76. 20957426 10.1007/s10488-010-0319-7 PMC3068522

[26] Glasgow R, Vogt T, Boles S. Evaluating the public health impact of health promotion interventions: The RE-AIM framework. Am J Public Health. 1999;89(9):1322–1327. 10.2105/AJPH.89.9.1322 10474547 PMC1508772

[27] Hwang S, Birken S, Melvin C, Rohweder C, Smith J. Designs and methods for implementation research: Advancing the mission of the CTSA program. J Clin Transl Sci. 2020;4(3):159–167. 10.1017/cts.2020.16 32695483 PMC7348037

[28] Pinnock H, Barwick M, Carpenter C, et al. Standards for reporting implementation studies (StaRI) statement. BMJ. 2017;356:i6795. 10.1136/bmj.i6795 28264797 PMC5421438

[29] Smith J, Li D, Rafferty M. The implementation research logic model: A method for planning, executing, reporting, and synthesizing implementation projects. Implement Sci. 2020;15(84):1–12. 10.1186/s13012-020-01041-8 32988389 PMC7523057

[30] Schouw D, Mash R, Ameh P, Fatusin B, Engmann S. Evaluating the implementation of online research training and mentorship among early-career family physicians in sub-Saharan Africa. Prim Health Care Res Dev. 2025;26(e3):1–11. 10.1017/S146342362400063X PMC11735121 39781644

